# Gender effects of agricultural cropping work and nutrition status in Tanzania

**DOI:** 10.1371/journal.pone.0222090

**Published:** 2019-09-06

**Authors:** Hitomi Komatsu, Hazel Malapit, Mysbah Balagamwala

**Affiliations:** 1 International Food Policy Research Institute, Washington, DC, United States of America; 2 Oxford Policy Management, Oxford, England, United Kingdom; University of Reading, UNITED KINGDOM

## Abstract

Although agriculture is an important source of food and income for food expenditures, women’s involvement in the agricultural cropping production process could increase their work load and reduce their BMI. Using three waves of the Tanzania National Panel Survey, we investigate the extent to which time spent in agricultural crop production affects women and men’s nutritional status among non-overweight individuals (age 20–65). We also test whether the impact of agricultural cropping work on nutritional status is modified by access to agricultural equipment, and whether gender differences exist. The study finds that time spent in agricultural cropping work is negatively associated with BMI for non-overweight individuals, albeit of small magnitude, and this finding is consistent across different crop production processes. This suggests that agricultural interventions should not ignore the implications of increasing work intensities on nutrition. While increased agricultural production could improve nutritional status by increasing agricultural income and food, the gains in nutritional status could be offset by an increase in work effort of doing agricultural work. Our results suggest that it is possible that access to equipment reduced effort for one production activity, but increased work for other activities in the production process, such as in harvesting. Furthermore, we find that the BMI of women in households with a hand powered sprayer is positively related to time spent in weeding, fertilizing, and non-harvest activities, while it is negatively correlated for men. It is possible that access to a hand powered sprayer may have helped reduce women’s work, for example, in weeding, while this was not the case for men’s work such as in ridging and fertilizing. Further disaggregation of agricultural activities in the dataset would have been helpful to provide more insights on the gender roles.

## Introduction

Agriculture is an important source of food and income in developing countries, especially for the poor [1], [2], [3]. In Tanzania, agriculture accounts for 26 percent of the GDP, and three quarters of the population [4]. Over 80 percent of economically-active women are employed in agriculture [4], [5]. However, agricultural work could also cause women to increase their work effort and lengthen working hours [2], [3]. If women spend more time on agricultural production, they may have less time to care for, or process and prepare nutritious food for themselves and their families. Certain agricultural activities involve exerting physical energy, and may be detrimental to women and men’s nutritional status [6], [2], [3].

While there is a growing body of literature on the linkages between agricultural production and nutrition, evidence on the effect of household agricultural production on women’s anthropometry is limited and so far inconclusive [1], [7], [8], [3]. Some find that although the nutrition effect of agricultural production exists for children, it does not exist for adults [9], [10]. For example, in Tanzania, higher crop output value and livestock ownership have a positive effect on children’s anthropometry, but there is no evidence of any impact on adult anthropometry [10]. Similarly, in Nepal, production diversity is positively associated with child anthropometry, but there is no effect on maternal anthropometry [9]. Lack of evidence of a direct link between agricultural production and women’s anthropometry may occur if agricultural work entails having to exert more energy. Therefore, while agricultural interventions may lead to increased caloric consumption or intake of micronutrient-rich foods [8], agricultural work could reduce BMI. It may explain why few studies have identified a direct effect of agricultural production on women’s nutritional status [11].

The purpose of this study is to investigate the extent to which time spent in agricultural crop production affects women and men’s nutritional status using three waves of the Tanzania National Panel Survey (NPS). We also test whether the impact of agricultural cropping work on nutritional status is modified by access to agricultural equipment, and whether there is a gender difference: does owning agricultural equipment increase or decrease the burden of agricultural work? If engaging in agricultural cropping work increases women’s work intensity and reduces nutritional status, then agricultural interventions need to be cognizant of these consequences.

We use an individual level fixed effects model to estimate the effect of time spent in agricultural cropping work on women and men’s BMI. We test whether ownership of agricultural equipment mediates the impact of agricultural work on nutritional status by interacting the time spent in work with ownership of agricultural equipment. Because crop production involves different stages of production with varying levels of work effort, we disaggregate the analysis by assessing the time spent on: total farm work; land preparation and planting; weeding, ridging, fertilizing and non-harvest activities; and harvesting.

## Agricultural work and nutrition

Authors in [3] outline six pathways through which agriculture can affect nutrition. Pathways 1 and 2 refer to the importance of agriculture as a source of food for own consumption, and a source of income for food and non-food expenditures, respectively. Pathway 3 argues that agricultural production can affect the relative prices of food. Pathway 4 highlights the role that agriculture plays on child and maternal nutrition by influencing women’s empowerment and intra-household decision-making. Yet, there could be nutritional trade-offs in increasing women’s engagement in agriculture. Working long hours in agriculture could reduce women’s time to prepare nutritious food and adequately care for their children and families. Pathway 5 suggests that the reduction in time for household chores or getting services (including health services) could compromise women’s own nutrition or that of their families. Further, pathway 6 posits that agricultural work could also involve arduous work that requires women to exert physical energy, leading to a reduction in women and children’s nutritional status. The focus of this paper is to test pathway 6, namely that that women and men’s time spent in farm work can affect their nutritional status.

Many studies suggest that higher energy expenditure from agricultural work has a negative effect on women’s nutritional status. In the Pune district of India, even though men’s involvement in farming has no relationship with their Body Mass Index (BMIs), there is a negative correlation for women [12]. Similarly, in India, female agricultural workers have lower BMIs than nonagricultural workers, after controlling for wealth, education, locality, and other individual and household characteristics [13]. In Andhra Pradesh (in India), body weight, body fat, basic metabolic rate, and energy intake of women working in agriculture are lower during the lean season compared to the harvest season, but this is not the case for women who do not work in the fields, implying that agricultural seasons affect energy expenditure and nutrition of agricultural workers [14]. A similar study on Tanzania estimates that women’s energy expenditure level is high in April due to the agricultural workload, while between July and October, female farmers experience the biggest weight gains when their energy expenditure declines [15]. Authors in [6] find that time spent in agricultural activities has a negative effect on women’s BMIs in Ghana.

However, not all agricultural tasks are equal in terms of energy costs. Rao et al [16] measure the energy costs of various farm work and domestic chores in a rural community in Pune, India, and find that weeding entails considerable high energy costs, similar to that of walking with a load of firewood. They estimate that some of the most tiring activities are grinding cereals on millstone and chopping firewood. According to [17], harvesting wheat, separating paddy, and milking involve higher energy expenditure than picking paddy or collecting fodder in India. Moreover, some tasks such as harvesting are seasonal while milking is a regular activity. Studies conducted in Malaysia and Gambia find the sowing of seeds to be the most energy-intensive farming activity conducted by women [18], [19].

The level of work effort could also differ by the type of agricultural tools used, and whether the tools are mechanized or not. The use of mechanized farm equipment could reduce energy costs and time required to carry out the task [20], [21], [22], [23]. Alternatively, the equipment could be heavy and bulky, and may actually increase energy costs even if it saves time. Therefore, the net effect on energy expenditure may be dependent on the type of machinery used. Yet, ownership, use, and control of farm equipment are determined by gender norms [24], [25], [26]. In Ethiopia and Kenya, [27] find that most households commonly reduce labor burden by hiring labor or using draft animals for land preparation activities–tasks that are usually undertaken by men. However, similar strategies to reduce labor are not commonly applied to tasks that women consider to be energy-intensive.

Access to agricultural equipment could affect crop production workload in several ways. On one hand, work burden might be lower–both in energy and time—when labor-saving equipment is used, and may lead to a positive impact on nutritional status. On the other hand, while new technologies may be time saving, they may not necessarily save energy if they are bulky, heavy, or difficult to use. Further, when new machineries are introduced, women’s work burdens could actually increase due to the gendered division of labor in the crop production process [25]. For example, if men are in charge of preparing the land, and clearing the fields is made easier with access to tractors, women may have to plant more seeds, or engage in more weeding or harvesting. Therefore, access to machineries could reduce effort for one production activity, but increase work for other activities in the production process [25], [28]. Household ownership of agricultural equipment may also lead to unequal access and control between women and men [27]. Authors in [27] cite several factors that limit women’s access to mechanized technology. These include norms, such as expectations on women to work hard, lack of access to information, the patriarchal nature of decision-making processes, and the lack of access to assets and services. Therefore, we cannot make assumptions about the mechanisms through which technologies affect nutrition *a priori* in this setting. Mechanization could also replace poor and unskilled agricultural laborers and reduce their incomes and nutritional status.

There are several factors that limit women’s ability to demand and adopt labor- and energy-saving technologies. Social norms impose expectations on women to work hard [27]. Additionally, women’s time is not valued highly and consequently, investment in such tools is not considered economically viable [25]. Women’s access to information on new technology is limited due to their time burden, relative lack of education and skills, lack of access to financial services, and due to patriarchal norms in intra-household decision making [27], [29], [26], [25]. While the design of tools is considered gender-neutral, many are not suitable for women as they are created for men’s physiques [26], and may be considered culturally-inappropriate [29].

Gender divisions in agricultural labor are dynamic and depend on the context and circumstances, so it is difficult to predict how the adoption of a new technology would impact individual welfare. According to a previous study in Tanzania, men are more likely to use mechanized equipment compared to women in farm work [30]. This differentiation was identified in other countries in another study [29]. Previous studies have also shown that when new technologies are introduced, men take over tasks that were previously assigned to women [25]. The shifting of responsibilities could impact intra-household labor allocations and resource reallocations due to the change in control over agricultural outputs [25].

In this paper, we hypothesize that time spent in agricultural cropping work could have a negative relationship with nutritional status. We also hypothesize that ownership of agricultural equipment could mediate the relationship between work and nutritional status, and expect gender differences in these relationships. However, we do not know, *a priori*, whether it could increase the negative relationship or reduce the negative effect.

## Data and methods

### Data

We use publicly available data from the Tanzania National Panel Survey (NPS), which was collected by the Tanzania National Bureau of Statistics and the Living Standards Measurement Study—Integrated Surveys on Agriculture (LSMS-ISA) in three waves: October 2008 –October 2009; October 2010 –November 2011; and October 2012 –November 2013 [31]. The Tanzania NPS, a national-level longitudinal study tracking the same households over time, included a sample of 3,265 households in the first wave, which grew to 3,786 households in the second wave, and 5,010 households in the third wave. Samples increased over time because the survey teams were able to track and interview households that split and grew. The household attrition rates were low at 3 percent for wave 2 and 4 percent for wave 3 [31]. The interviews were conducted in different months spread out throughout the year. The survey collects information on socio-economic characteristics, height and weight for all household members, and includes a detailed agricultural module administered to the household members that manage and/or own each plot [32]. The agricultural module collects individual-disaggregated labor input at the plot-level by task and by season, namely, the long rainy season (March-May) and the short rainy season (November and December). The labor input data reports the number of days each household member spent on: 1) land preparation and planting; 2) weeding, ridging, fertilizing and non-harvest activities; and 3) harvesting during the long rainy season or the short rainy season. The practice of selecting the plot manager or owner as the respondent for the specific plot-level questions is based on the assumption that he or she is the most knowledgeable person about all activities regarding the plot. This is a standard approach to collecting agricultural data, used by the LSMS-ISA surveys [32]. However, like all surveys that rely on the most knowledgeable household member as the respondent, this approach may result in measurement errors due to imperfect information regarding other household members’ activities. This approach may also be subject to social desirability bias, for example, underreporting women’s labor inputs in tasks that are deemed socially unacceptable. In the case of Tanzania, we cannot say *a priori* which direction of bias is likely to occur.

We restrict the analysis sample to agricultural households, defined as those that cultivated any plot in the last year. About 3 percent of households in the sample did not cultivate on any plots during last agricultural seasons, and these non-agricultural households are excluded.

We calculate the number of days a household member spent in each activity in the last year in the following way. First, an individual’s labor inputs are aggregated by agricultural activity for each wave of the panel. Second, if the value for labor input is missing for a particular household member X, but labor inputs are recorded for other members of the household, then the household member X’s labor contribution is assumed to be zero. Thirdly, if the total number of days recorded for a particular individual across activities exceeds 365 days, then that individual’s labor input for all activities are replaced by missing values. The approach of dropping observations with total labor input that exceeds 365 days is consistent with the approach taken by authors in [32], who studied the female share of agricultural labor using LSMS/ISA data in Tanzania. This resulted in dropping 246 observations. Outliers (top 1 percent) for each agricultural activity domain are also replaced by missing values.

We dropped 12 observations that had biologically implausible BMI values, defined as values less than 12 kg/m^2^, or greater than 70 kg/m^2^ [33]. Pregnant and lactating women (827 women) are excluded from the estimations. Since the survey does not ask about women’s pregnancy or lactating status, proxy variables are created for both. Women are assumed to be lactating if their child is 6 months old or younger. We calculate whether women were pregnant based on the interview date and youngest child’s age in the subsequent wave. For women in wave 3, we use the child’s age information from wave 4 (2014–2015). If women’s pregnancy status cannot be obtained this way because of attrition in the subsequent wave, we exclude these women (2,734 women) from the analysis. We also exclude overweight individuals in the estimations to focus on individuals who may be at risk of becoming underweight from doing agricultural work. Overweight individuals (1,068 individuals) comprise 10.3 percent of men and 26.2 percent of women.

We use an individual fixed effects model, which requires individuals to be in the sample for at least two waves [34]. Trimming the sample provides a pooled wave-person sample of 3,786 men and 1,727 women who are aged 20–65 years, approximately 87 percent of whom live in rural areas. Table 1 presents the sample size by year and sex.

**Table 1:** Number of observations by year and sex (individuals who are age 20–65).

*Wave (Year)*	*Men*	*Women*
**1 (2008/2009)**	1,042	698
**2 (2010/2011)**	1,437	754
**3 (2012/2013)**	1,307	275
***Total***	*3*,*786*	*1*,*727*

Source: Authors’ calculations using Tanzania NPS/LSMS-ISA.

Table 2 gives the summary statistics for key variables for women and men aged 20–65 who are not overweight (i.e. BMI is below 25 kg/m^2^). Adult BMI was calculated by dividing the weight in kilograms by the square of the height in meters. Ownership of agricultural equipment was measured at the household level, and was collected in each wave. The summary statistics disaggregated by each wave are given in S1 Table. S2 Table provides the summary statistics of independent variables not listed in Table 2 below.

**Table 2:** Summary statistics (pooled wave-person data).

	Men	Women	Test of means
	n = 3,786	n = 1,727	
**Dependent variable**			
Body Mass Index	20.8	21.0	*
**Explanatory variables**			
**Days worked in cropping production**			
Total farm work (days in last year)	59.6	58.8	
Total land preparation and planting (days in last year)	21.9	21.5	
Weeding, fertilizing and non-harvest (days in last year)	21.6	21.4	
Total harvesting (days in last year)	16.1	15.9	
**Other control variables**			
Responsible for keeping large livestock (= 1, 0 otherwise)	23.2%	14.9%	***
Responsible for keeping goats or sheep (= 1, 0 otherwise)	25.6%	15.1%	***
Responsible for keeping chicken, turkey, rabbits, or pigs (= 1, 0 otherwise)	33.9%	61.3%	***
Responsible for collecting water (= 1, 0 otherwise)	15.3%	64.6%	***
Responsible for collecting firewood (= 1, 0 otherwise)	9.6%	33.3%	***
Household owns seed planter (= 1, 0 otherwise)	11.5%	8.4%	***
Household owns hand powered sprayer (= 1, 0 otherwise)	7.6%	5.6%	***
Household owns tractor (= 1, 0 otherwise)	2.7%	1.0%	***
Land cultivated or owned (acres)	8.40	6.25	***
**Men age 19+ lives in household**	100%	80.6%	***

Authors’ calculations using Tanzania NPS/LSMS-ISA.

***p<0.01

* p<0.1. Household weights used.

Women’s BMI is slightly higher than men’s. Men spent more time (59.6 days) than women (58.8 days) in total farm work and in land preparation and planting, although the gender difference is not statistically significant. The gender difference in the average time spent weeding, fertilizing and non-harvest activities is also not statistically significant. In a separate study, [28] found that, in the Eastern, Southern, and Lake zones in Tanzania, men were largely responsible for land clearing and ridging in preparation for planting, whereas women were responsible for planting and weeding, especially for cassava. It is possible that the separation of the time spent in land preparation from planting, and the separation of weeding from ridging in the questionnaire would make the gender division of labor more apparent in our data, although there are variations across regions, and the division of labor is not static [30].

Livestock maintenance and fetching water and firewood have implications on physical work effort, and the responsibilities are determined by gender roles. Men are more likely to be responsible for keeping large livestock (cows, bulls, calves or heifers) and small livestock (goats and sheep), while women tend to be responsible for keeping poultry. Women (65 percent) disproportionately bear the burden of collecting water, and almost a third of women are responsible for collecting firewood versus only 10 percent of men. Approximately, 19 percent of women live in households with no adult male, which could potentially increase their workload. Women are more likely to live in households with fewer agricultural equipment and with smaller plots of land.

### Empirical methodology

The endogeneity between agricultural work and nutrition status is of particular concern. On one hand, unobservable characteristics of individuals, such as people’s values, attitudes or social norms that affect time allocation decisions, may also impact nutritional status. On the other hand, causality here may be reversed—namely that nutritional status or health can impact labor productivity because workers who are healthier can work longer hours and produce more [35], [10]. This endogeneity concern exists only with respect to the weight of individuals, and not their height, because our sample includes adults aged 20–65 years who are unlikely to grow taller.

One way to address this problem is to use an instrumental variable technique by finding instruments that affect time allocation in agriculture, and not nutritional status directly. However, it is difficult to find valid instruments that meet these criteria. For example, [10], argues that rainfall data, which is often used as instruments, affects not only agricultural production, but it can also adversely affect health status through an increase in the incidence of infectious diseases. Other potential instruments, such as the use of irrigation and soil quality, are also problematic in that there are few households with access to irrigated plots, and problems in measuring soil quality [10].

Unobserved characteristics could be constant, or time varying. To account for time-invariant unobserved characteristics, an individual fixed effects (within) regression model can be used to remove individual time-invariant characteristics. These unobserved characteristics not only include values and norms that an individual believes in, but also account for the underlying health status and physical traits that do not change over time.

To account for time varying health shocks, such as when a person gets sick or has an injury, a dummy variable is included indicating whether the person visited a health provider in the last four weeks.

The exclusion of overweight individuals in the estimations is another way to account for reverse causality. Conceptually, losing weight is a good nutritional outcome for overweight individuals, so pooling overweight and non-overweight individuals clouds the analysis. Aside from simplifying the interpretation of our findings, excluding overweight individuals also eliminates the possibility that being overweight prevents an individual from engaging in agricultural work. As a robustness check, we estimated the model for the sample including overweight individuals, and the coefficients on time spent are slightly larger, but the results are generally similar to the subsample without overweight individuals. Women and men are pooled in the estimations because we want to see whether there are gender differences in the impact of work on nutritional status.

We estimate the following nutrition production function (1) using an individual fixed effects (within) model for women and men aged 20 and 65:

$$BMI_{it}=\beta_{0}+\beta_{1}\;time_{ait}+\beta_{2}\;asset_{t}+\beta_{3}\;time_{ait}\;x\;woman_{i}+\beta_{4}\;time_{ait}\;x\;asset_{t}+\beta_{5}\;asset_{t}\;x\;woman_{i}+\beta_{6}\;time_{ait}\;x\;asset_{t}\;x\;woman_{i}+\beta_{7}\;X_{it}+\beta_{8}\;w_{t}+v_{i}+u_{it}$$ (1)

where *BMI*_*it*_ is the BMI of individual *i* at time *t*, *β*_*i*_ are parameters to be estimated, *time*_*ait*_ is the natural log of the number of days (plus 0.01) spent in agricultural activity *a* in the previous year, *woman*_*i*_ is equal to 1 if individual is a woman, *X*_*it*_ is the individual and household characteristics at time *t*, *w*_*t*_ is a dummy variable for the year of the interview, *v*_*i*_ is the time-invariant individual characteristics, and *u*_*it*_ is the idiosyncratic error term. Since error terms of individuals in the same households are likely to be correlated, we allow for this correlation by clustering the standard errors by household. The term *asset*_*t*_ is equal to 1 if household owned a particular agricultural asset at time *t*. The agricultural assets are those that could potentially save time or reduce workload and comprise of dummy variables indicating whether the household owned a tractor, seed planter, or a spraying machine. The choice of the agricultural equipment to include in the regression is determined by which equipment is likely to impact which activity. For example, time spent in land preparation and planting is likely to be affected by the use of tractors and seed planters. The use of hand powered sprayers is likely to impact the time spent in weeding and fertilizing.

Our coefficients of interest are the terms *β*_*1*_ to *β*_*6*_, which estimate the extent to which time spent in crop production, ownership of agricultural machinery, and sex of the household member, and their interactions impacts nutrition. Using fixed effects estimation on (1) eliminates the individual level time invariant error term, *v*_*i*_ [36].

Individual and household characteristics at time *t* include age, whether the household head is a woman, marital status, household composition by age and sex. We exclude variables that change little over time such as education, head of household’s education and location specific indicators such as distance to markets, whether the household is in a rural area, and regions because they are eliminated from the transformation of the fixed effects model [37].

An individual’s responsibility in collecting water, firewood, or keeping livestock entails different work intensities. A study in rural India reports that drawing water from a well, carrying two water containers on the head, walking with a load of firewood, and chopping firewood involve significant energy costs compared to, for example, cleaning an animal shed [16]. Donor funded water investment projects reduced the time burden of women responsible for collecting water [38]. Since the difference in work intensity in domestic chores and livestock maintenance is likely to affect nutritional status, we include dummy variables indicating whether the person is responsible for collecting water, firewood, for keeping large livestock, small livestock (goats or sheep), and pigs or poultry.

The ability to hire nonfamily labor could reduce family members’ workload and increase production. A study evaluating a dairy intensification program aimed at women, find that the intervention had increased the workload for women in households producing a medium level of output as compared to higher intensity households as the former were not able to hire additional labor [39]. Therefore, we include a dummy variable indicating whether the household hired labor in the last season.

Ownership of cattle, goats, sheep, pigs, or chicken is expected to affect nutrition by providing access to eggs, milk and meat products [3]. Using (organic or inorganic) fertilizers or pesticides could increase agricultural productivity and thereby improve nutrition. Both the above are included in the analysis.

Two sets of variables represent the household’s socioeconomic status: the size of land cultivated or owned by the household (in acres) and real per capita food consumption expenditure (in log form). For the latter, we use the per capita food consumption expenditure calculated by the National Bureau of Statistics of Tanzania, which is estimated from the quantity and value of food consumed that is bought, produced themselves, or given as gifts [31]. Fisher food price indices were used to adjust for spatial and temporal price differences [31].

In addition, we include indicators on access to improved water, sanitation, and electricity because not only do they represent the household’s wealth, access to these assets could save time. Access to improved water and sanitation also directly contributes to improved health and nutrition [3].

The month of the interview is included to control for the agricultural seasons, which have implications on work intensity, and for the fluctuation in calorie intake between lean and harvest seasons. In Tanzania, agricultural cropping workload is expected to be greater in rainy seasons (from February to May) when there is more agricultural activity [15]. February is also a lean season where there is less calorie intake [15].

There are several limitations to the study. First, in the survey, the time of reference for the agricultural activity is only recorded in the number of days. Yet, the length of the day (in hours) could vary by agricultural work. Typical work hours were only collected beginning in wave three of the NPS (2012/2013), so it was not possible to examine the effect of the number of hours worked in this study. Second, it is not clear whether cropping activities include post-harvest activities, which women are more likely to be responsible for [30]. Ignoring post-harvest activities is likely to underestimate women’s overall agricultural work burden. Third, the ownership or use of agricultural equipment is recorded at the household level and not at the individual level, therefore it is not clear whether an individual used the equipment or not even when it is available at the household. Lastly, because the data does not contain individual level food consumption, we control for per capita food consumption expenditure. While these variables do not control for unequal intra-household food distribution, a recent study on rural farm households in Western Kenya showed that household-level and individual-level dietary indicators are positively correlated, suggesting that household level food consumption expenditure data is a good proxy for individual food consumption [40].

## Results and discussion

Coefficients of farm work and its interaction effects from the individual fixed effects estimations of nutritional status for women and men are given in Table 3. Columns 1, 2, 3 and 4 present the impact on BMI of the days spent in: (1) total farm work; (2) land preparation and planting; (3) weeding, fertilizing and non-harvest activities; and (4) harvesting in the past short and long rainy season. Control variables are included in the regressions but are not shown in Table 3; they are presented in S3 Table.

**Table 3:** Results summary: Estimating women and men’s body mass index, age 20–65 using individual fixed effects model.

	Dependent variable: BMI			
	(1) Total work	(2) Land preparation and planting	(3) Weeding and fertilizing	(4) Harvesting
ln of total agricultural work	-0.033***			
	(0.009)			
ln of land preparation and planting		-0.030***		
		(0.009)		
ln of weeding and fertilizing			-0.036***	
			(0.009)	
ln of harvesting				-0.023***
				(0.009)
Hand powered sprayer	0.327*		0.313***	
	(0.167)		(0.121)	
Tractor	-0.108	-0.296		-0.071
	(0.164)	(0.253)		(0.124)
Seedplanter		0.139		
		(0.122)		
Hand powered sprayer x tractor	0.187			
	(0.281)			
Seedplanter x tractor		-0.029		
		(0.271)		
Woman x total work	0.036*			
	(0.021)			
Hand powered sprayer x total work	-0.027			
	(0.041)			
Woman x hand powered sprayer	-0.553*		-0.470**	
	(0.335)		(0.222)	
Woman x sprayer x total work	0.024			
	(0.079)			
Tractor x total work	-0.029			
	(0.032)			
Woman x tractor	-0.617*	-0.320		-0.373*
	(0.367)	(0.328)		(0.211)
Woman x tractor x total work	0.026			
	(0.087)			
Hand powered sprayer x tractor x total work	-0.005			
	(0.058)			
Woman x hand powered sprayer x tractor	-0.446			
	(0.895)			
Woman x hand powered sprayer x tractor x total work	0.405*			
	(0.230)			
Woman x land preparation and planting		0.029		
		(0.019)		
Seedplanter x land preparation and planting		-0.018		
		(0.031)		
Woman x seedplanter		-0.129		
		(0.200)		
Woman x seedplanter x land preparation and planting		0.005		
		(0.061)		
Tractor x land preparation and planting		0.151***		
		(0.055)		
Woman x tractor x land preparation and planting		-0.198**		
		(0.080)		
Seedplanter x tractor x land preparation and planting		-0.106		
		(0.072)		
Woman x seedplanter x tractor		-0.341		
		(0.451)		
Woman x seedplanter x tractor x land preparation and planting		0.383***		
		(0.143)		
Woman x weeding and fertilizing			0.038*	
			(0.019)	
Sprayer x weeding and fertilizing			-0.024	
			(0.037)	
Woman x sprayer x weeding and fertilizing			0.013	
			(0.062)	
Woman x harvesting				0.027
				(0.018)
Harvesting x tractor				-0.035
				(0.028)
Woman x tractor x harvesting				0.019
				(0.075)
Observations	5,513	5,513	5,513	5,513
R-squared	0.041	0.038	0.040	0.035
Number of individuals	2,543	2,543	2,543	2,543
**Testing for significance of interaction effects:**	**F-statistic**			
Work x woman x equipment is equal to zero	3.11*	7.2***	0.05	0.06
Work x woman is equal to zero	3.05*	2.31	3.85*	2.14
Woman x equipment is equal to zero	0.25	0.57	4.58**	3.13*
Work x equipment is equal to zero	0.01	2.17	0.42	1.49

Authors’ calculations using Tanzania NPS/LSMS-ISA. Overweight individuals, pregnant or lactating women are excluded. Control variables are included in the estimations and the results for the other control variables are shown in S3 Table. Standard errors clustered by households are in parentheses.

***p<0.01

** p<0.05

* p<0.1.

The results indicate that time spent in agricultural cropping work is significantly negatively correlated with BMI. This is consistent even when agricultural activities are disaggregated into different tasks. However, it should be noted the magnitude of the effect is quite small. For example, a 10 percent increase in total farm work reduces BMI by 0.003 (from column 1 in Table 3). This suggests that engaging in agricultural cropping work reduces BMI but at a small magnitude. An alternative model without interaction effects (not shown) resulted in similar coefficients for time spent in agricultural cropping in Table 3.

To test whether ownership of agricultural equipment modifies the impact of work on BMI, we use a Wald test to see whether the interaction terms (between farm work, ownership of agricultural equipment, and sex of household member) are jointly equal to zero. The results (in last 4 rows of Table 3) indicate that the interaction terms of work, ownership of equipment, and being a woman are significant in model 2 (land preparation and planting). The interaction effects of ownership of equipment and being a woman is also significant in model 3 (weeding and fertilizing).

**Fig 1:**
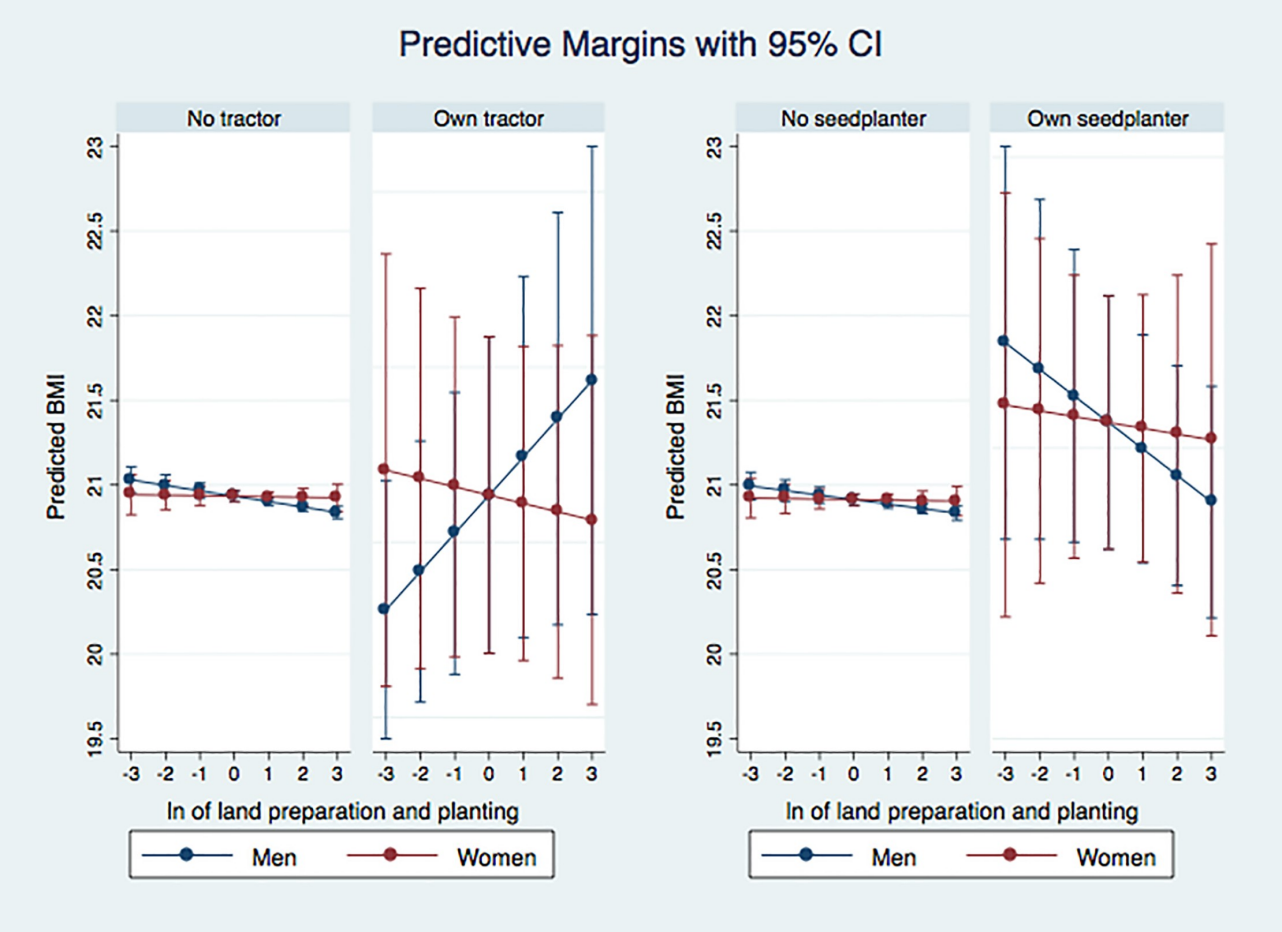
Estimating the effect of land preparation and planting on women and men’s body mass index, age 20–65. Authors’ calculations using Tanzania NPS/LSMS-ISA.

It is difficult to see whether the ownership of equipment mitigates or increases the effect of work by looking at Table 3 alone because of the multiple interaction effects. To visualize how agricultural equipment modifies the effect of farm work on nutritional status, we plot the predictive margins on BMI at different levels of land preparation and planting, by ownership of equipment and the sex of the individual in Fig 1. The 95 percent confidence intervals are also shown in Fig 1. The graph on the far right represents individuals in households with a seed planter, and the graph to its immediate left includes individuals without. The two graphs on the left-hand side show the BMI disaggregated by whether the household owns tractors.

BMI of farmers with seed planters falls more rapidly with work than the BMI of those without, implying that using seed planters increases the negative effects of engaging in land preparation and planting. The ownership of seed planters seems to have higher energy costs as it negatively correlated with BMI for both men and women.

In contrast, the ownership of tractors tends to mitigate the impact of engaging in land preparation and planting for men, but not for women. The BMI of men in households with tractors is positively related to time spent in land preparation and planting. The BMI of women with tractors is negatively related to this work, but the size of the effect is small. One of the reasons for the positive relation between land preparation and planting and BMI for male farmers with tractors could be that access to tractors reduces the work burden of this activity while this may not be the case for women. A second possible reason could be that individuals with tractors are more likely to be small commercial farmers and are less likely to be subsistence farmers. Our descriptive statistics in Table 4 show that households with tractors cultivate much larger land than those without. According to [21], the use of tractors in Sub-Saharan Africa tends to be restricted to the small commercial farm sector. Even though the size of land and wealth proxies are included as covariates in the regression, it may not have sufficiently captured the wealth advantages small commercial farmers have over subsistence farmers. Therefore, the ownership of tractors could represent lower work burdens in land preparation and planting especially for men, and greater wealth. It should be noted that the 95 percent confidence intervals for the farmers with seed planters or tractors are larger than those without, which is likely because of the smaller sample size of households with the equipment, leading to larger margins of errors.

**Table 4:** Size of land owned or cultivated (in acres) by ownership of equipment.

	Owns Tractor		Owns seed planter		Owns hand powered sprayer	
	No	Yes	No	Yes	No	Yes
Land size (acres)	7.2	31.7	6.9	15.0	7.2	14.0
Number of observations	5372	141	4988	525	5162	351

Authors’ calculations using Tanzania NPS/LSMS-ISA.

**Fig 2:**
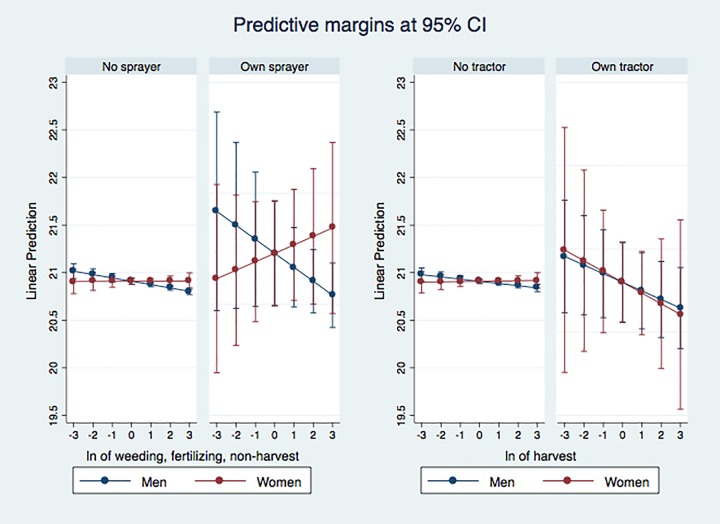
Estimating the effect of weeding and fertilizing, and harvesting on women and men’s body mass index, age 20–65. Authors’ calculations using Tanzania NPS/LSMS-ISA.

The estimations for the effects of weeding and fertilizing, and harvesting on BMI can be seen in Fig 2.

Ownership of a hand powered sprayer seems to increase the negative effect of working on weeding, fertilizing, and non-harvest activities on men’s nutritional status from the left-hand side graph, but the reverse is true for women. The BMI of women in households with hand powered sprayer is positively related to time spent in these activities. Previous research in Tanzania shows that weeding and non-harvest activities tend to fall in women’s domain [28], and ridging and fertilizing tend to fall into men’s responsibilities [30], [28].

Our results suggest that access to hand powered sprayer may have helped reduce women’s work in weeding, while this was not the case for men’s work in ridging and fertilizing. Unfortunately, the data does not disaggregate these activities into two groups (weeding versus ridging and fertilizing), which would have helped give us more insights into the gender division of labor and the role that equipment may have played in reducing workloads.

According to the graph on the right-hand side, ownership of tractors increases the negative effect of harvesting on BMI. Since farmers with tractors worked on larger farms, they are possibly producing higher yields and have an increasing workload.

## Conclusion

Our results show that time spent in farm work is negatively correlated with nutritional status for non-overweight individuals, albeit of small magnitude. This implies that agricultural interventions should not ignore the implications of work intensities on nutrition. The improvement in nutrition from increased agricultural production may be offset by the increase in work effort required by agricultural work.

Unfortunately, there are few datasets that contain information on labor inputs, other agricultural inputs, production, and nutrition of household members. In order to empirically test the six pathways outlined in the agriculture-nutrition pathways [3], it is recommended that agricultural datasets include information on nutrition, and that production data contain information on agricultural inputs, including labor.

The BMI of farmers with certain agricultural equipment (such as a seed planter, hand-powered sprayer) decreased at a faster pace with time spent in crop production. However, while the BMI of male farmers with tractors had a positive correlation with time spent in land preparation and planting, it was negatively correlated with time spent harvesting. It is possible that access to machineries reduced effort for one production activity, but increased work for other activities in the production process, such as in harvesting. The BMI of women in households with hand powered sprayer is positively related to time spent in weeding, fertilizing, and non-harvest activities, while it is negatively correlated for men. Our results suggest that access to hand powered sprayer may have helped reduce women’s work in weeding, while this was not the case for men’s work in ridging and fertilizing but further disaggregation of activities in the dataset would have been helpful to understand this better.

Increasing women and men’s access to mechanized farming tools could reduce women’s energy exertion and improve their nutritional status. But it is important to consider the impact it could have on time and energy costs of not just one production activity but of other activities. The introduction of new equipment should also account for the physical differences between women and men, and its implications on work burdens. For example, ergonomically-designed equipment catering to the women’s needs can reduce their workload and improve productivity [41]. When new technologies are introduced, men might take over the tasks previously assigned to women [25]. It is imperative to ensure that women do not lose control over the proceeds of the agricultural products they were previously responsible for. Further, mechanization could displace farmers who are poor and low skilled and consequently reducing their incomes and nutritional status.

Further research on efficiently measuring energy requirements, especially in large surveys in developing countries, will add to the growing evidence base on this topic. Measuring the intensity, duration, and frequency of time use for different activities, in addition to individual-level food consumption, will be key to accurately capturing energy expenditure [42].

## Supporting information

S1 Table Summary statistics by wave. (PDF)

S2 Table Descriptive statistics of control variables not listed in Table 2 (pooled wave-person data). (PDF)

S3 Table Coefficient estimates of control variables that were not shown in Table 3. Estimating women and men’s body mass index using individual fixed effects model.
(PDF)
