# Supplementary material for: Gender effects of agricultural cropping work and nutrition status in Tanzania
Source: PLoS One. 2019 Sep 6;14(9):e0222090. doi: 10.1371/journal.pone.0222090 (PMC6730922; doi:10.1371/journal.pone.0222090)
Supplement: S1 Table — (PDF) [file pone.0222090.s001.pdf]

S1 Table: Summary Statistics by wave

	Wave 1			Wave 2			Wave 3		
	Men n= 1,042	Women n= 698	Test of means	Men n= 1,437	Women n= 754	Test of means	Men n= 1,307	Women n= 275	Test of means
**Dependent variable**									
Body Mass Index	20.8	20.8		20.8	21.0	*	20.8	21.1	**
**Explanatory variables**									
**Days worked in cropping production**									
Total farm work (days in last year)	58.3	56.7		59.3	58.6		61.2	66.2	
Total land preparation and planting (days in last year)	22.8	22.0		21.6	20.9		21.4	21.9	
Weeding, fertilizing and non-harvest (days in last year)	20.2	20.4		21.2	21.0		23.4	25.8	
Total harvesting (days in last year)	15.2	14.3		16.4	16.7		16.4	18.5	
**Other control variables**									
Responsible for keeping large livestock (=1, 0 otherwise)	22.0%	15.2%	***	22.9%	15.2%	***	24.7%	12.9%	***
Responsible for keeping goats or sheep (=1, 0 otherwise)	24.8%	12.3%	***	24.7%	14.7%	***	27.6%	25.8%	
Responsible for keeping chicken, turkey, rabbits, or pigs (=1, 0 otherwise)	41.2%	65.1%	***	31.9%	58.8%	***	29.5%	58.1%	***
Responsible for collecting water (=1, 0 otherwise)	17.1%	66.2%	***	15.2%	64.5%	***	13.8%	60.4%	***
Responsible for collecting firewood (=1, 0 otherwise)	10.1%	31.0%	***	10.0%	35.9%	***	8.7%	31.6%	***
Household owns seed planter	12.5%	8.6%	***	10.8%	7.6%	***	11.4%	10.8%	
Household owns hand powered sprayer	7.9%	5.5%	***	8.3%	5.7%	**	6.4%	5.5%	
Household owns tractor	2.6%	1.0%	***	2.7%	1.1%	***	2.9%	0.3%	***
Log of land cultivated or owned	1.02	0.97	**	0.89	0.85		0.91	0.91	
**Men age 19+ lives in household**	100%	80.1%	***	100%	80.9%	***	100%	80.6%	***

Authors' calculations using Tanzania NPS/LSMS-ISA. ***p<0.01, ** p<0.05, * p<0.1. Household weights used
