# Supplementary material for: Gender effects of agricultural cropping work and nutrition status in Tanzania
Source: PLoS One. 2019 Sep 6;14(9):e0222090. doi: 10.1371/journal.pone.0222090 (PMC6730922; doi:10.1371/journal.pone.0222090)
Supplement: S2 Table — (PDF) [file pone.0222090.s002.pdf]

S2 Table: Descriptive statistics of control variables not listed in Table 2 (pooled wave-person data)

	Men n= 3,786	Women n= 1,727	Test of means
**Demographic information**			
Age	39.2	40.2	***
Married (monogamous or polygamous)[1]	63.3%	60.6%	
Visited a health provider in the last 4 weeks[1]	11.4%	15.4%	***
**Household characteristics**			
Lives in a female headed household[1]	7.1%	29.0%	***
Age of household head	45.6	47.7	***
Number of girls age 0-4	0.56	0.45	***
Number of boys age 0-4	0.53	0.44	***
Number of girls age 5-10	0.60	0.56	
Number of boys age 5-10	0.56	0.56	
Number of girls age 11-18	0.64	0.60	
Number of boys age 11-18	0.64	0.67	
Number of women 19-59	1.19	1.24	**
Number of men 19-59	1.41	0.94	***
Number of women 60 +	0.11	0.15	**
Number of men 60 +	0.15	0.17	**
**Agriculture**			
Received advice about agricultural activities last year[1]	59.1%	51.1%	***
Inorganic fertilizer used in last agricultural season[1]	15.2%	16.9%	
Organic fertilizer used in last agricultural season[1]	26.3%	22.4%	***
Pesticide used in last agricultural season[1]	14.7%	13.5%	
Hired labor in last agricultural season[1]	42.8%	39.2%	***
**Assets, consumption, and housing characteristics**			
Log of per capita food consumption, real	12.7	12.6	***
Household owned cows, bulls, calves, heifers in last 12 months[1]	31.9%	26.6%	***
Household owned goats in last 12 months[1]	37.0%	32.6%	***
Household owned sheep in last 12 months[1]	12.7%	12.8%	
Household owned pig, chicken, turkey, or rabbits in last 12 months[1]	70.5%	70.9%	
Drinking water from safe sources[1]	35.7%	36.3%	
Has improved toilet (flush, VIP, or improved pit latrine)[1]	10%	9%	
Electricity or solar as major fuel for lighting[1]	7%	6%	

Authors' calculations using Tanzania NPS/LSMS-ISA. ***p<0.01, ** p<0.05, * p<0.1. Household weights used.
[1] denotes dummy variables.
