# Supplementary material for: Gender effects of agricultural cropping work and nutrition status in Tanzania
Source: PLoS One. 2019 Sep 6;14(9):e0222090. doi: 10.1371/journal.pone.0222090 (PMC6730922; doi:10.1371/journal.pone.0222090)
Supplement: S3 Table — Estimating women and men’s body mass index using individual fixed effects model. (PDF) [file pone.0222090.s003.pdf]

S3 Table: Coefficient estimates of control variables that were not shown in Table 3: Estimating women and men's body mass index using individual fixed effects model

	Dependent variable: BMI			
	(1) Total work	(2) Land preparation and planting	(3) Weeding and fertilizing	(4) Harvesting
Married (monogamous or polygamous)	0.054	0.048	0.055	0.056
	(0.061)	(0.062)	(0.061)	(0.062)
Lives in a female headed household	-0.092	-0.081	-0.088	-0.078
	(0.144)	(0.145)	(0.145)	(0.143)
Age of head of household	-0.022	-0.024	-0.021	-0.022
	(0.022)	(0.023)	(0.023)	(0.022)
Age of head of household squared	0.000	0.000	0.000	0.000
	(0.000)	(0.000)	(0.000)	(0.000)
Age	0.098**	0.099**	0.098**	0.095**
	(0.045)	(0.045)	(0.045)	(0.045)
Age squared	-0.001***	-0.001***	-0.001***	-0.001***
	(0.000)	(0.000)	(0.000)	(0.000)
Number of girls age 0-4	-0.001	-0.007	-0.005	-0.006
	(0.037)	(0.037)	(0.037)	(0.037)
Number of boys age 0-4	-0.018	-0.019	-0.016	-0.009
	(0.039)	(0.039)	(0.039)	(0.039)
Number of girls age 5-10	-0.069*	-0.069*	-0.074**	-0.072*
	(0.038)	(0.038)	(0.037)	(0.038)
Number of boys age 5-10	0.006	0.002	0.002	0.005
	(0.038)	(0.039)	(0.038)	(0.038)
Number of girls age 11-18	-0.037	-0.037	-0.040	-0.042
	(0.039)	(0.039)	(0.038)	(0.039)
Number of boys age 11-18	-0.040	-0.035	-0.039	-0.035
	(0.041)	(0.040)	(0.040)	(0.041)
Number of women 19-59	0.007	0.013	0.008	0.002
	(0.043)	(0.044)	(0.043)	(0.043)
Number of men 19-59	0.025	0.035	0.022	0.038
	(0.044)	(0.044)	(0.044)	(0.043)
Number of women 60 +	-0.000	-0.023	0.002	-0.021
	(0.118)	(0.117)	(0.117)	(0.117)
Number of men 60 +	0.005	0.004	0.002	-0.001
	(0.107)	(0.108)	(0.106)	(0.108)
Visited a health provider in the last 4 weeks	-0.080	-0.081	-0.078	-0.078
	(0.056)	(0.057)	(0.055)	(0.056)

Received advice about agricultural activities last year	0.033	0.034	0.031	0.031
	(0.042)	(0.042)	(0.042)	(0.042)
Household owned cows, bulls, calves, heifers in last 12 months	-0.030	-0.044	-0.047	-0.034
	(0.089)	(0.090)	(0.089)	(0.089)
Household owned goats in last 12 months	0.043	0.045	0.045	0.037
	(0.081)	(0.082)	(0.082)	(0.082)
Household owned sheep in last 12 months	0.246***	0.254***	0.246***	0.261***
	(0.093)	(0.093)	(0.094)	(0.094)
Household owned pig, chicken, turkey, or rabbits in last 12 months	0.072	0.066	0.067	0.061
	(0.066)	(0.066)	(0.066)	(0.066)
Responsible for keeping large livestock	-0.026	-0.017	-0.006	-0.022
	(0.087)	(0.089)	(0.088)	(0.086)
Responsible for keeping goats or sheep	-0.041	-0.040	-0.037	-0.037
	(0.079)	(0.080)	(0.079)	(0.079)
Responsible for keeping chicken, turkey, rabbits, or pigs	-0.066	-0.063	-0.063	-0.059
	(0.048)	(0.049)	(0.048)	(0.049)
Responsible for collecting water	-0.007	-0.012	-0.008	-0.009
	(0.049)	(0.049)	(0.049)	(0.049)
Responsible for collecting firewood	0.008	0.009	0.003	0.008
	(0.052)	(0.052)	(0.052)	(0.052)
Inorganic fertilizer used in last agricultural season	0.105	0.110	0.109	0.113
	(0.081)	(0.081)	(0.081)	(0.081)
Organic fertilizer used in last agricultural season	0.038	0.033	0.036	0.024
	(0.052)	(0.052)	(0.052)	(0.052)
Pesticide used in last agricultural season	-0.041	-0.055	-0.039	-0.046
	(0.058)	(0.058)	(0.059)	(0.058)
Hired labor in last agricultural season	-0.009	-0.012	-0.012	-0.009
	(0.038)	(0.038)	(0.038)	(0.038)
Log of land cultivated or owned (acres)	0.003	0.003**	0.003*	0.003*
	(0.002)	(0.001)	(0.002)	(0.001)
Drinking water from safe sources	0.102**	0.098**	0.103**	0.097**
	(0.048)	(0.047)	(0.047)	(0.047)
Has improved toilet (flush, VIP, or improved pit latrine)	0.008	0.003	0.013	0.005
	(0.068)	(0.067)	(0.068)	(0.068)
Electricity or solar as major fuel for lighting	-0.084	-0.075	-0.088	-0.078
	(0.090)	(0.091)	(0.089)	(0.091)
Log of food consumption expenditure p.c.	0.018	0.018	0.017	0.021
	(0.041)	(0.041)	(0.041)	(0.041)
Dummy variable for the month of survey included?	Yes	Yes	Yes	Yes

Dummy variable for the year of the survey included?	Yes	Yes	Yes	Yes
Constant	19.549***	19.549***	19.553***	19.504***
	(1.615)	(1.618)	(1.618)	(1.613)
Observations	5,513	5,513	5,513	5,513
R-squared	0.041	0.038	0.040	0.035
Number of individuals	2,543	2,543	2,543	2,543

Authors' calculations using Tanzania NPS/LSMS-ISA. Overweight individuals, pregnant or lactating women are excluded. The rest of the results are shown in Table 3. Standard errors clustered by households are in parentheses. ***p<0.01, ** p<0.05, * p<0.1.
